# Dissecting the influence of Neolithic demic diffusion on Indian Y-chromosome pool through J2-M172 haplogroup

**DOI:** 10.1038/srep19157

**Published:** 2016-01-12

**Authors:** Sakshi Singh, Ashish Singh, Raja Rajkumar, Katakam Sampath Kumar, Subburaj Kadarkarai Samy, Sheikh Nizamuddin, Amita Singh, Shahnawaz Ahmed Sheikh, Vidya Peddada, Vinee Khanna, Pandichelvam Veeraiah, Aridaman Pandit, Gyaneshwer Chaubey, Lalji Singh, Kumarasamy Thangaraj

**Affiliations:** 1 CSIR-Centre for Cellular and Molecular Biology, Uppal Road, Hyderabad, India; 2 Theoretical Biology and Bioinformatics, Utrecht University, Utrecht, Netherlands; 3 Evolutionary Biology Group, Estonian Biocentre, Tartu, Estonia

## Abstract

The global distribution of J2-M172 sub-haplogroups has been associated with Neolithic demic diffusion. Two branches of J2-M172, J2a-M410 and J2b-M102 make a considerable part of Y chromosome gene pool of the Indian subcontinent. We investigated the Neolithic contribution of demic dispersal from West to Indian paternal lineages, which majorly consists of haplogroups of Late Pleistocene ancestry. To accomplish this, we have analysed 3023 Y-chromosomes from different ethnic populations, of which 355 belonged to J2-M172. Comparison of our data with worldwide data, including Y-STRs of 1157 individuals and haplogroup frequencies of 6966 individuals, suggested a complex scenario that cannot be explained by a single wave of agricultural expansion from Near East to South Asia. Contrary to the widely accepted elite dominance model, we found a substantial presence of J2a-M410 and J2b-M102 haplogroups in both caste and tribal populations of India. Unlike demic spread in Eurasia, our results advocate a unique, complex and ancient arrival of J2a-M410 and J2b-M102 haplogroups into Indian subcontinent.

Population history of India has been under investigation to study the earliest settlement of anatomically modern humans Out of Africa and later to study the demographic episodes of Eurasia^1^,^2^,^3^. Late Pleistocene heritage of modern humans in India has been supported by archaeological findings of Middle Palaeolithic tools^4^. Mitochondrial genetic studies agree upon deep rooting Late Pleistocene maternal ancestry of the Indian subcontinent^1^,^5^,^6^. However, some Y-chromosomal studies argue that Indian subcontinent displays considerable genetic relatedness to West Eurasia corresponding to recent events^7^,^8^. First settlement of modern humans in India was 60–70 KYA (thousands years ago)^6^,^9^,^10^. In early Holocene, South Asia was a continent of hunter-gatherers. During and after Neolithic period, agriculturists dominated the land, especially the fertile river valleys^11^. Emergence of agriculture led to the major socio-cultural transition and technological development in human prehistory. The oldest evidence of agriculture comes from the Fertile Crescent (~11 KYA), the centre for demic diffusion^12^. However, evidences of first agriculture from South Asia indicate a timeline (~9-10 KYA) closer to the emergence of agriculture in the Fertile Crescent^13^.

Previous studies have established that J2a-M410 and J2b-M102 represent the Y-chromosomal component associated with demic diffusion of Neolithic farmers in North Africa and Eurasia from Mesopotamia (Iraq and Syria)^14^,^15^,^16^. The J2-M172 has been associated with different cultures and populations in history, such as Mediterranean/Aegan^15^, Greco-Anatolian, Mesopotamian and Caucasian. Presence of J2a-M410 and J2b-M102 in India has been considered a result of gene influx from Western Asia^17^,^18^. Worldwide spatial distribution of haplogroup (HG) J2a-M410 coincides with presence of archaeological records of painted pottery and ceramic figurine culture^19^,^20^. Similar material culture dating ~9 KYA has been recovered from the Neolithic sites of Mehrgarh located West of Indus Valley (now in Pakistan)^21^.

The population of the sub-continent shows an outstanding biological and cultural variation, which has been shaped by geographical, ecological, social and linguistic factors^22^. Many recent studies, particularly from the field of evolutionary genetics, have shown the extent to which each of these factors has contributed to the generation and maintenance of this diversity. It is pertinent that paternal gene pool of India comprises mainly HGs of autochthonous origin of Late Pleistocene ancestry^17^,^18^,^23^, and received very little gene flow from outside^7^,^8^. Many Y chromosomal studies of India addressing major demographic questions have included and broadly discussed HG J2-M172^17^,^18^,^23^. However, the arrival and distribution of HG J2-M172 subclades in India have not been studied comprehensively yet. Hence, the aim of this study is to address the following: (1) whether or not, the demic diffusion model stands true for the current distribution of J2-M172 in India in the backdrop of independent origin of agriculture in South Asia; (2) which contemporary populations have the closest affinities with J2-M172 in India; (3) whether distribution of J2a-M410 in India is more dominant in castes than in tribes^18^; and (4) what social, linguistic or geographical factors might have influenced the distribution of J2-M172 subclades. To address the above, we investigated the presence of different subclades of HG J2-M172 in diverse set of Indian populations and their affinities with rest of the world.

## Results

### J2-M172 composition in India: two brother clades

Out of 3023 samples, 355 chromosomes belonged to J2-M172 HG (Supplementary Table S1 and Supplementary Table S2). We could not find any J*-M304 and J2*-M172 chromosome in India. We report, a rare presence of 7 chromosomes belonging to J1-M267 from different populations which most likely entered into the Indian subcontinent in historical time^24^. In this study, we did not find any J2a1b-M67 Y-chromosome. This makes Indian J2a-M410 pool unique from the rest of the world, as majority of the J2a-M410 pool consists of J2a1b-M67 Y-chromosomes (Supplementary Table S1). Similarly, we could not find any J2a1a-M47, and J2a1h1-M158 chromosomes, which represent minority of J2a-M410 pool outside India. Genotyping Z2396, a newly discovered SNP, has divided the Indian J2a-M410 pool into two groups: derived and ancestral for Z2396, proving it to be polyphyletic in India. Our study shows a frequent presence of M68, a rare marker, in various tribes and groups of South India suggests its autochthonous origin. Presence of M68 was also reported in few samples of Indian origin in a study from Southeast Asia^25^. J2b-M102/M12 HG in India comprises largely of J2b2-M241 chromosomes with minor presence of J2b1-M205 in northwest (NW) region. Occurrence of one J2a1a-M47 and two J2a1h1-M158 chromosomes have been reported earlier in Indian samples^18^,^23^, though we could not find any in the present study.

### Phylogeography of J2a-M410 and J2b-M102

**Figure 1:**
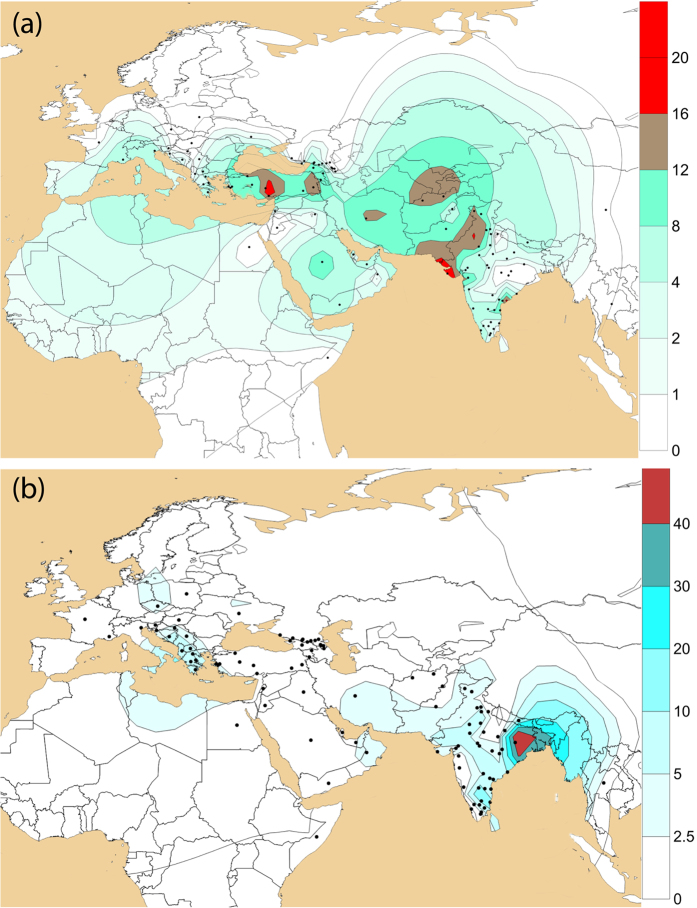
Contour maps showing worldwide geographical frequency distribution of haplogroups. (**a**) J2a-M410 and (**b**) J2b-M102. The maps were generated using Surfer8 of Golden Software (Golden Software Inc.), following the Kriging procedure. Black dots indicate sampling locations.

**Figure 2:**
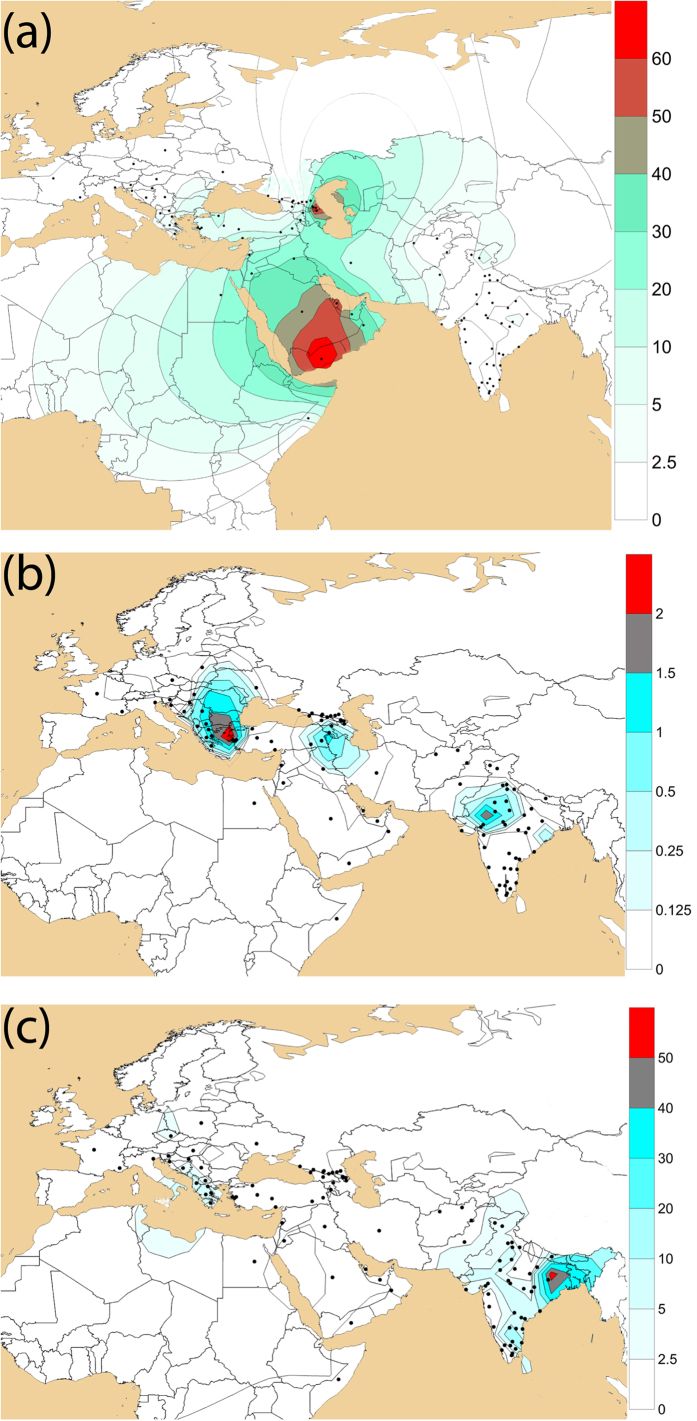
Contour maps showing worldwide geographical frequency distribution of haplogroups. (**a**) J1-M267, (**b**) J2b1-M205 and (**c**) J2b2-M241. The maps were generated using Surfer8 of Golden Software (Golden Software Inc.), following the Kriging procedure. Black dots indicate sampling locations.

We found moderate occurrence of J2a-M410 and J2b-M102 (0–8%) in different populations inhabited in different parts of India (Supplementary Table S1). We observed substantial high frequencies of J2a-M410 (17–50% in Toda, Chenchu, Banjara, Kamboj, Lohana and Kashmiri Muslims etc.) and J2b-M102 (15–35% in Asur, Narikuravar, Pichakuntla, Shikari and Mondi, etc.) in several populations. J2a-M410 is mainly concentrated towards the NW border of India (comprising Gujarat and Rajasthan). However, high frequency and variance of J2a-M410 in PTGs (primitive tribal groups) like Toda (trasitional pastoralists) and Chenchu (hunter-gatherers and foragers) invoke interesting insights. Contrary to earlier belief, predominant presence and high variance of J2a-M410 among remote tribes dismisses any caste-specific distribution of J2a-M410 in India^18^. We found the distribution of both the clades geographically pronounced. From West Asia, J2a-M410 wave seems to expand West towards South-eastern Europe and East towards Central Asia and eventually to South Asia (Fig. 1a). Dense focal points of J2a-M410 can be seen along the northwest border of South Asia reaching up to South India. However, it shows a drastic decline towards East of India, consistent with our previous study^26^. Unlike J2a-M410, J2b-M102 is concentrated in Eastern India (Fig. 1b). Worldwide frequency patterns of J1-M267, J2a-M410, J2b-M102, J2b1-M205 and J2b2-M241 indicates their unique history of distribution (Figs 1 and 2). Frequencies and Y-STR haplotype data of different subclades of J2-M172 are given in Supplementary Table S1, Supplementary Table S3 and Supplementary Table S4.

In NW and South India we found the highest variance and oldest TMRCA (time of most recent common ancestor) of J2a-M410 haplotypes, whereas in North, West and Central India the TMRCA (Table 1, Supplementary Table S5 and Supplementary Table S6) was comparatively recent. Similar to J2a-M410, J2b-M102 shows the oldest TMRCA in NW region and younger TMRCA in Central, southern, northern and eastern India. Conversely, J2b-M102 shows a slightly different distribution and younger TMRCA than its brother clade J2a-M410 in India (Table 1, Supplementary Table S5 and Supplementary S6). The J2b-M102 variance (Supplementary Table S5) is in contrast with its geographical distribution, suggesting a strong founder effect in East India.

Among linguistic groups, our results indicate that Indo-Europeans (IE) and Dravidians (DR) have received the J2a-M410 influx approximately at the same time. However, our results indicate a very late influx and rare presence of the J2a-M410 HG in Austroasiatics (AA). In eastern region, J2b-M102 exhibits few dense focal points in contour map due to high frequency among some AA groups like Asur caused by founder effect. Comparing the TMRCA and diversity of J2b-M102 haplotypes between different linguistic groups (Table 1, Supplementary Table S5 and Supplementary Table S6) suggests that either AA received the gene flow from IE or DR very late or they have lost the diversity due to drift, followed by founder effect. Thus, the distribution of this haplogroup in India is governed primarily by geography. Apart from geographical separation, linguistic affiliation seems to be responsible to shape J2-M172 pool in India. Male effective population sizes for J2a-M410 show rapid expansion when moving from NW region to Gangetic plains while it drops rapidly in Central India and again expands in South India (Table 1, Supplementary Table S6 and Supplementary Fig. S1). However, J2b male effective population sizes remain more or less constant throughout the subcontinent except for East India, where it declines sharply (Table 1, Supplementary Table S6 and Supplementary Fig. S1).

### Affinities with world population

**Figure 3:**
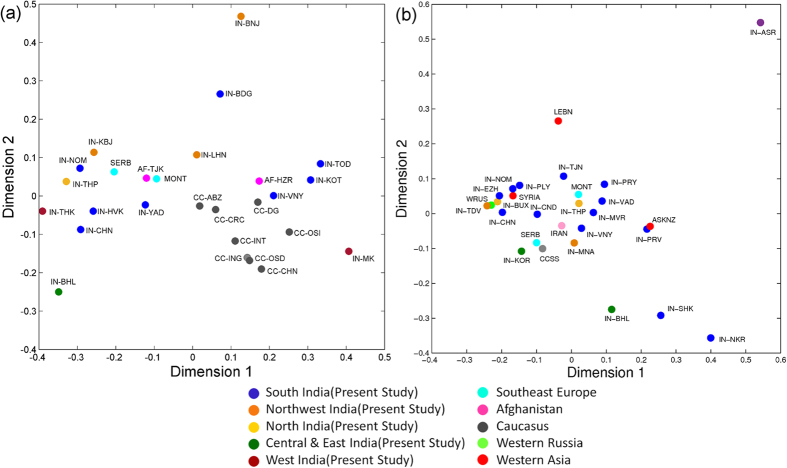
Multidimensional scaling (MDS) plot based on Rst values of Y-STRs for haplogroups. (**a**) J2a-M410 and (**b**) J2b-M102. Dots represent different populations worldwide. CC = Caucasian, IN = Indian, AF = Afghanistani, ABZ = Abkhaz, BDG = Baduga, BNJ = Banjara, BHL = Bahelia, CHN = Chechen (Chechnya), DG = Chechen (Dagestan), INT = Chechen Ingushetia, CHN = Chenchu, CRC = Circassians, HVK = Havik, HZR = Hazara, ING = Ingush, KBJ = Kamboj, KOT = Kota, LHN = Lohana, MK = Mahadev Koli, MONT = Montenegrin, OSD = Ossets (Digor), OSI = Ossets (Iron), NOM = Indian Nomads (Pichakuntla & Mondi), SERB = Serbian, TJK = Tajik, THK = Thakar, THP = Thapa, TOD = Toda, VNY = Vanniyar, YAD = Yadav, IRAN = Iranian, LEBN = Lebanese, MONT = Montenegrin, SERB = Serbian, SYRIA = Syrian, WRUS = WesternRussia, CCSS = Caucasus, ASR = Asur, BHL = Bahelia, BUX = Buxas, CND = CapeNadar, CHN = Chenchu, EZH = Ezhava, KOR = Korku, MVR = Maravar, MNA = Meena, NKR = Narikuravar, PLY = Paliyan, PRV = Paravar, PRY = Parayar, NOM = Indian Nomads (Pichakuntla & Mondi), SHK = Shikari, TDV = Tadvi, TJN = Tamil Jains, THP = Thapa, VNY = Vanniyar, VAD = Yadhava, ASKNZ = Ashkenazi.

**Figure 4:**
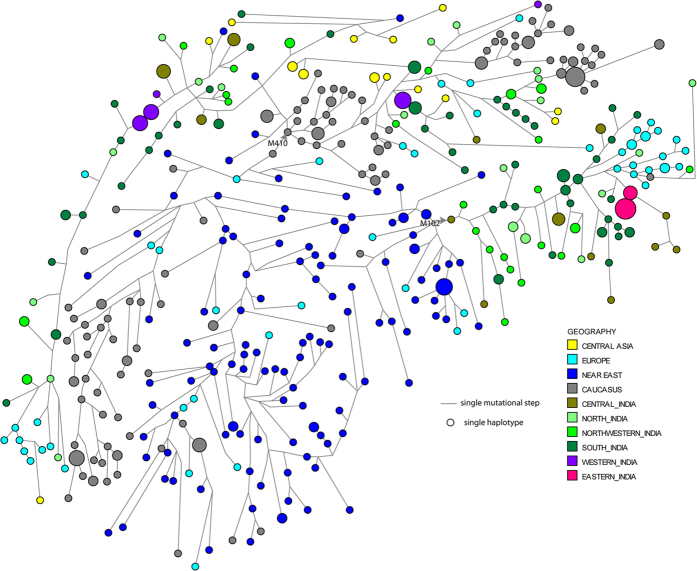
Network connecting Y-STR haplotypes within J2-M172. The network was constructed using a median joining with maximum parsimony (MP) algorithm as implemented in the Network 4.612 program. The size of the circle is proportional to the numbers of the samples.

MDS (Multidimensional Scaling) plot based on Rst values for HG J2a-M410 shows that Indian populations do not form a single cluster but are closer to the Central Asian and European populations, while Caucasians (populations from Caucasus) form a separate cluster (Fig. 3a). However, MDS for HG J2b-M102 shows that only few Indian nomadic tribes and East Indian populations lie outside the major Indian cluster (Fig. 3b). MJ (median joining) tree (Fig. 4) depicts geographically pronounced clustering of Y-STR haplotypes. No sharing of STR haplotypes for J2a-M410 and J2b-M102 along with geographical clustering has been observed. High diversities of J2a-M410 and J2b-M102 haplotypes are evident from the MJ and RM (reduced Median) networks (Fig. 4, Supplementary Fig. S2 and Supplementary Fig. S3).

## Discussion

J2-M172 is a predominant HG in West and Central Asia. Populations living West from India show high frequency, subclade variation and presence of paragroups. Worldwide subclade diversity and distribution of J2a-M410 suggest its spread from West and Central Asia into India through NW corridor (Fig. 1a). The spatial distribution of J2a-M410 throughout Middle East and Central Asia is overlapped by presence of Neolithic artifacts such as painted pottery and ceramic culture^19^. The earliest precursor known of Indus Valley civilization, Mehrgarh (NW of Indian subcontinent, now in Pakistan), provides one of the oldest (~9KYA) evidences of origin of agriculture and plant domestication suited by early Holocene climate^27^. Additionally, these Neolithic sites of Mehrgarh showed the earliest evidence of transformation of subsistence from hunting-gathering to settled agriculture owing to the idea that the first farmers from Indus were agro-pastoral, and semi-nomadic people^28^. It is interesting to note that the concentration of J2a-M410 over the geography largely mimics the agricultural centres^29^. J2a-M410 in India peaks at NW region and shows a clinal pattern towards Central and East, however, again rises considerably in South. J2b-M102 has been found in all parts of India in low to moderate frequency, but it is significantly frequent among some nomadic PTGs of South India. From eastern region, some of the Austroasiatic tribes carry a high frequency of J2b2-M241. In the present study, we also see high frequency of J2a-M410 and J2b-M102 in remote undisturbed foragers with recent history of hunting gathering (eg. Asur, Chenchu), pastorals (eg. Toda tribe with high J2a-M68) and nomadic tribes (eg. Banjara, Bahelia etc). Most of the nomadic tribes were from NW region or had recently migrated from the region towards South India (eg. Narikruwar, Shikari, Mondi, Pichakuntla). Considering these facts and arguments, one can deduce that these groups could be the relic of agro-pastoral communities spreading from the NW region of the subcontinent in the past.

Various studies have given evidences to support the influence of Neolithic from Near East on Indian subcontinent (in Mehrgarh) dated around 10.5 KYA 30 and references therein. Noted similarities between Mehrgarh and Near East are domesticated wheat varieties, early phases of farming, pottery, other archaeological artefacts, some domesticated plants and herd animals^30^. A vast arid region of Iran and Afghanistan lies in between Near East and Indus Valley, leaving possibility of rainfall agriculture only in the foothills and *cul-de-sac* valleys^31^. Yet, the area was not an undefeatable geographical barrier for Neolithic spread. Some sections of the Silk Road (route South of the Caspian sea) connecting Badakhshan (north-eastern Afghanistan and south-eastern Tajikistan) with West Asia, Egypt and India were in use by 5 KYA^32^. Other section of Silk Road connecting Badakhshan to the Mesopotamian plains (the Great Khorasan Road) was in use by 6 KYA^31^. Archaeological evidences support similarities among widely separated Neolithic sites in these regions^33^ and plausibility of migration of population^34^.

The AMOVA results found that geographically distant populations have higher Fst values (Table 2). Lower Fst values between NW India and geographical regions West from India show them to be less differentiated. Higher Fst values between NW and South India suggest their high level of differentiation. TMRCAs of J2a-M410 and J2b-M102 of NW and South India advocate an early arrival of these lineages to Indian subcontinent. Genetic relatedness of NW Indians with population to West from NW border and remarkable presence of J2-M172 HGs in remote Indian tribes along with other social strata, may represent the early Holocene expansion in NW India (including the Indus Valley) diffusing towards Central Asia and spreading agriculture eastwards to the Gangetic plains during pre-Harappan times (6-7 KYA). However, presence of J2-M172 subclades in India can not only be substantiated by Neolithic spread. Firstly, lack of any sublineage of J2a-M410 (M67, M47 and M92) representing majority of the pool outside India, implies towards an older and unique history of this HG into the subcontinent. J2a1b-M67 and J2a-M92 lineages have been well correlated temporally and spatially with the spread of earliest farmers and Bronze Age cultures in Anatolia, south-eastern Europe and Mediterranean^15^. Secondly, in indigenous Indian populations, negligible presence of other Y-HGs like R1b1b2-M269^35^, G2a-P15 and E1b1b1a1b-V13^16^,^36^,^37^, which are associated with demographic spread of Neolithic. Thirdly, in addition to prominent absence of J2a1b-M67 and J2a-M92 in southwestern Asia, practical absence of HGs J1-M267 and G-M201 in India, respectively, occur at 9% and 10.9% in Turkey^38^, 33.1% and 2.2% in Iraq^14^, and 3.4% and 6% in Pakistan^18^, indicate towards different dispersal events from Middle East to southeastern Europe and southwestern Asia. Complete absence of any paragroup of J clade in India reinforces the established theory of J2 subclades entering into the subcontinent from NW/West to India. Given all that, and granted their exogenous origin, J2a-M410 and J2b-M102 in the subcontinent may reflect any combination of unknown and known movements. Though, the genealogical ages for Indian J2a-M410 and J2b-M102 are correlating with appearance of agriculture in Indus Valley (~6KYA) and Mehrgarh (~9KYA) and falling well within the Neolithic range, differential presence and distribution of J2-M172 sublineages and other associated HGs depict a complex picture. Most likely events responsible for the current distribution of J2-M172 sublineages into Indian subcontinent could be any combination of 1) entry of herders from West and Central Asia/Middle East during late glacial maximum (LGM) of Holocene, 2) Neolithic demic diffusion from the West, and 3) Bronze and Iron age migration/admixtures.

## Conclusion

Absence or negligible presence of classical markers of Eurasian demic diffusion in India advocates against it to be the sole explanation of J2a-M410 and J2b-M102 distribution in the subcontinent. High variance, haplotype diversity with no sharing haplotype, geographically pronounced phylogeny and seemingly autochthonous origin of sublineage J2a-M68, suggest towards the antiquity of the HGs. Dispersal of J2a-M410 and J2b-M102 from Near East to NW region and further eastwards of the subcontinent seems to have unique and complex history of various known and unknown possible events. Regardless of the complexity of dispersal, NW region appears to be the corridor for entry of these haplogroups into India. Remarkable presence of J2a-M410 among tribal groups inhabited in remote geographical regions strongly dismisses the earlier belief of it to be caste-specific.

## Material and Methods

### Sample composition

We studied a total of 3023 Y- chromosomes belonging to 77 different Indian populations. Of these, 1102 individuals were from different primitive tribes, 362 individuals were from nomadic tribes, 856 individuals were from different castes and 541 individuals were from different tribes. These individuals were affiliated to different linguistic groups (~1249 Indo-Europeans, ~1163 Dravidians, ~407 Austroasiatics and ~78 Tibeto-Burman) distributed all over India. In addition to Indian samples, we also analysed 39 samples of Thapa population from Nepal. Details of populations studied are given in the Supplementary Table S2. Prior to the sample collection, informed written consents were obtained from all the subjects participating in this study. This study was approved by the Institutional Ethical Committee of the CSIR-Centre for Cellular and Molecular Biology, Hyderabad, India. The methods were carried out in accordance with approved guidelines.

### Y chromosome analysis

Biallelic Y chromosome markers defining HG J-M304 and its subclades (M304, M267, M172, M410, M102, M47, M67, M68, M158, M205, M241, Z2396 and Z1827) were genotyped following the hierarchy of the Y chromosome phylogeny. Samples were genotyped through Sanger sequencing using BigDye™ Terminator cycle sequencing kit (Applied Biosystems, USA). For Y-STR genotyping, every second or third sample from each population was randomly selected based on the frequency of the subclades of HG J-M304 in the respective population. Out of 355 J-M304 possessing Y chromosomes, 158 were genotyped for 17 Y-STR markers with AmpF*l*STR^®^ PCR amplification kit (Applied Biosystems, USA). Detailed method of genotyping is given in Text S1. J2-M172 Individuals were resolved for downstream biallelic markers and assigned to the HG J2-M172 subclades (ISOGG, http://www.isogg.org/tree/).

### Haplotype analysis

We obtained haplogroup frequency data for 6966 individuals from different studies, representing various geographical regions and ethnicity worldwide^14^,^18^,^38^,^39^,^40^,^41^,^42^,^43^,^44^,^45^,^46^,^47^,^48^,^49^,^50^,^51^. We also retrieved worldwide dataset of 17 Y-STR markers for 1157 individuals belonging to HG J2-M172 for comparison^23^,^26^,^39^,^51^,^52^,^53^,^54^,^55^,^56^,^57^. Details of the dataset are given in Supplementary Table S1 and Supplementary Table S4. Median joining (MJ) and reduced median (RM), resolved with the maximum parsomony (MP) algorithm, were created using the 15 Y-STR data (Supplementary Table S3 and Supplementary Table S4) and Network 4.612 software (www.fluxus-engineering.com). Equal weights were given to each locus. One Steiner tree was selected and shown (Fig. 1, Supplementary Fig. S2 and Supplementary Fig. S3). The age estimates (Supplementary Table S5) based on 15 Y-STR loci variations (Supplementary Table S3, Supplementary Table S4 and Supplementary Table S7) were calculated using the method described in Zhivotovsky *et al.* 58 and updated in Sengupta *et al.* 18. The TMRCA of Y chromosomal HGs have been derived from *ρ* statistic, assuming evolutionary mutation rate 6.9 × 10^−4^ per locus per generation^58^ as well as TD statistics, assuming genealogical mutation rate 2.1 × 10^−3^ per locus per generation^59^,^60^,^61^.

Population diversity indices, Rst values and AMOVA (Analysis of Molecular Variance) based F*st* were calculated using Arlequin 3.5 software^62^. MDS (Multi Dimensional Scaling) plots of Rst values were drawn using MATLAB 8.03 (http://www.mathworks.nl/products/matlab/). HG isofrequency maps were generated using Surfer v 8 (Golden Software Inc., Golden, Colorado), following the Kriging procedure. We excluded two DYS385 loci from all current analyses due to technical issue. We have also filtered out some highly deviating haplotype samples from any age estimation analyses. The deviating samples were detected by determining their high mutational distance from the modal haplotypes. The selected samples used for TMRCA analyses are listed in Supplementary Table S7.

### BATWING analysis

TMRCAs and the times of individual population splits were calculated using the Bayesian approach implemented in BATWING, considering exponential growth from a constant-size population model^63^. We used broad prior distributions: gamma (2, 400) for population growth rate per generation (α), gamma (1, 200) for the time in coalescent units when exponential growth began, normal (2000, 1000) for effective population size (N)^35^. To achieve more plausible time estimates for Neolithic coalescent sublineage like J2-M172^64^, we used genealogical rates. We considered three different sets of mutation rates and prior distributions for each marker^65^,^66^ (Supplementary Table S8). For each BATWING simulation, we performed 300 million MCMC cycles. We discarded first 50 million MCMC iterations as burn-ins and sampled 1 in every 500 cycle for estimation. Thus, the results are inferred from 500,000 samples taken from 250 million MCMC iterations. The TMRCA was calculated as the product of ‘N’, generation time (30 years) and the height of the tree ‘T’ estimated by BATWING (Table 1 and Supplementary Table S6). The statistical analysis was performed using R package 3.2.0 (http://www.R-project.org/).

## Additional Information

**How to cite this article**: Singh, S. *et al.* Dissecting the influence of Neolithic demic diffusion on Indian Y-chromosome pool through J2-M172 haplogroup. *Sci. Rep.* **6**, 19157; doi: 10.1038/srep19157 (2016).

## Supplementary Material

Supplementary Table

Supplementary Information

## Figures and Tables

**Table 1:** BATWING results of time of most recent common ancestor (TMRCA) and effective population size for groups of geographically categorized populations using (1) “observed” mutation rates^66^, (2) “observed” mutation rates^65^ (3) mutation rates predicted from logistic model^65^ for J2a-M410 and J2b-M102 Y-STRs.

	TMRCA (KYA)			N (Effective Population Size)			TMRCA (KYA)			N (Effective Population Size)		
	Median	2.5%	97.5%	Median	2.5%	97.5%	Median	2.5%	97.5%	Median	2.5%	97.5%
	J2a-M410						J2b-M102					
Worldwide												
(1)	6.0	2.9	12.6	796	563	1132	3.3	1.6	8.5	420	301	590
(2)	7.1	3.6	16.2	842	613	1142	4.5	2.2	10.9	579	428	784
(3)	9.9	4.6	24	1153	819	1609	6.3	3.1	15.7	807	595	1092
Northwest India												
(1)	3.7	1	14.6	179	96	346	3.2	0.7	15.8	202	101	443
(2)	5.1	1.4	19.7	248	137	475	4.5	1	21.1	281	143	601
(3)	7.1	1.9	27.1	345	189	660	6.2	1.4	29.5	389	197	835
North India												
(1)	3.1	0.7	14.3	236	119	509	2.5	0.4	16.1	129	55	351
(2)	4.3	1	19.5	331	169	703	3.5	0.6	21.6	180	78	477
(3)	6	1.4	26.5	457	233	956	4.8	0.8	30.1	252	108	671
South India												
(1)	4.1	1.6	12.8	281	176	459	2.3	0.7	8	120	69	212
(2)	5.7	2.2	17.3	395	257	623	3.2	1	10.7	168	98	289
(3)	8	3	23.7	545	351	852	4.4	1.4	14.9	233	136	403
Central India												
(1)	2.2	0.3	14.5	45	18	121	2.6	0.5	13.6	116	55	269
(2)	3.1	0.5	19.7	62	26	166	3.6	0.8	18.2	161	77	366
(3)	4.3	0.7	27.1	87	35	229	5	1	25.3	225	108	512
East India												
(1)	—	—	—	—	—	—	0.2	0	1.9	4	0	16
(2)	—	—	—	—	—	—	0.2	0	2.7	5	1	22
(3)	—	—	—	—	—	—	0.3	0	3.7	7	1	30
Central Asia												
(1)	4.9	1.6	16	290	175	500	—	—	—	—	—	—
(2)	6.7	2.3	21.2	392	246	648	—	—	—	—	—	—
(3)	9.2	3.2	29.4	546	339	904	—	—	—	—	—	—
Caucacus												
(1)	3.8	2.1	8.6	446	338	588	—	—	—	—	—	—
(2)	5.3	2.7	11.1	585	455	733	—	—	—	—	—	—
(3)	7.4	3.6	16.3	799	579	1020	—	—	—	—	—	—
Europe												
(1)	3.3	0.5	23.9	175	72	521	2.3	0.5	10.9	69	34	145
(2)	4.6	0.7	32.1	248	103	702	3.1	0.7	14.6	96	48	200
(3)	6.3	1	42.9	345	144	951	4.3	0.9	20.3	134	66	278

**Table 2:** Analysis of Molecular Variance (AMOVA) using (a) J2a and (b) J2b Y-STR between groups of geographically categorized populations.

Geographical Group	Fst
(a)	
Northern & North-western India vs Europe	0.1546
Northern & North-western India vs Central Asia	0.18
Northern & North-western India vs Caucasus	0.3747
Northern & North-western India vs Southern India	0.3493
Southern India vs Central Asia	0.304
Southern India vs Europe	0.3315
Southern India vs Caucasus	0.4104
(b)	
Northern & North-western India vs Europe	0.5008
Northern & North-western India vs Near East	0.5591
Northern & North-western India vs Central India	0.5463
Northern & North-western India vs Southern India	0.5763
Northern & North-western India vs Eastern India	0.695
Southern India vs Europe	0.5738
Southern India vs Near East	0.6201
