# Supplementary material for: Dissecting the influence of Neolithic demic diffusion on Indian Y-chromosome pool through J2-M172 haplogroup
Source: Sci Rep. 2016 Jan 12;6:19157. doi: 10.1038/srep19157 (PMC4709632; doi:10.1038/srep19157)
Supplement: Supplementary Information [file srep19157-s2.pdf]

Supplementary Information

Dissecting the influence of Neolithic demic diffusion on Indian Y-chromosomal pool through J2-M172 haplogroup

Sakshi Singh[1], Ashish Singh[1], Raja Rajkumar[1], Katakam Sampath Kumar[1], Subburaj Kadarkarai Samy[1], Sheikh Nizamuddin[1], Amita Singh[1], Shahnawaz Ahmed Sheikh[1], Vidya Peddada[1], Vinee Khanna[1], Pandichelvam Veeraiah[1], Aridaman Pandit[2], Gyaneshwer Chaubey[3], Lalji Singh[1,4], Kumarasamy Thangaraj[1*]

1 CSIR-Centre for Cellular and Molecular Biology, Uppal Road, Hyderabad, India
2 Theoretical Biology and Bioinformatics, Utrecht University, Utrecht, Netherlands
3 Evolutionary Biology Group, Estonian Biocentre, Tartu, Estonia
4 Genome Foundation, Hyderabad, India

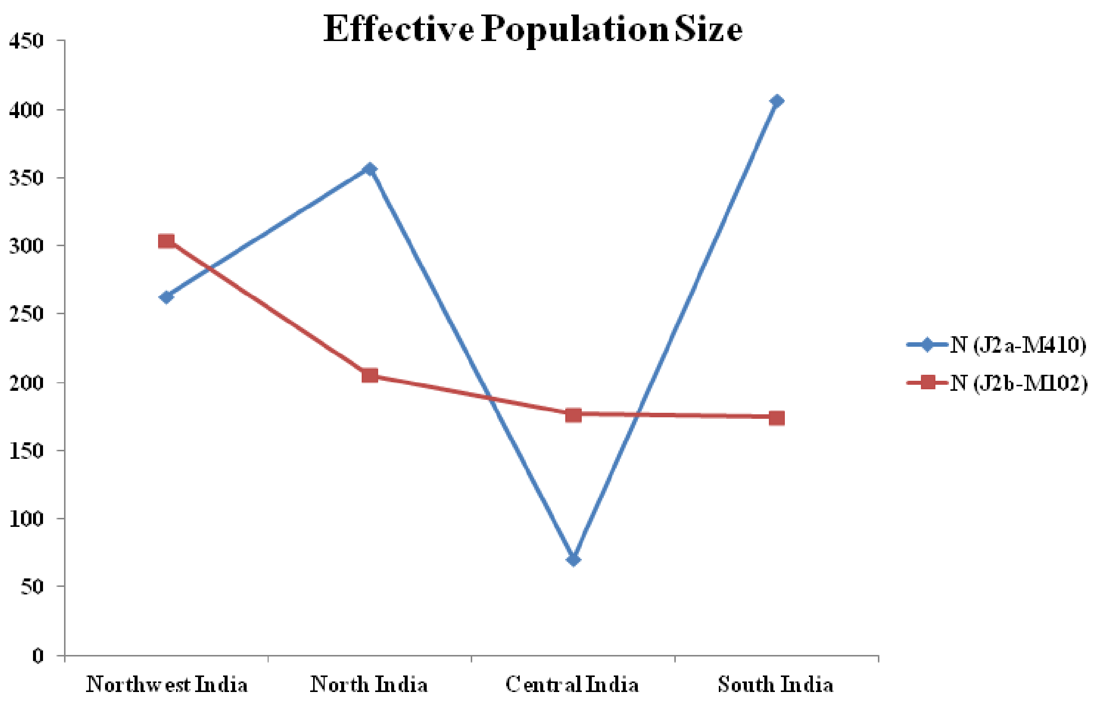

Figure S1. Effective population sizes for different geographical regions in India for J2a-M410 and J2b-M102 haplogroups. The effective population sizes were calculated using BATWING. N= Number of individuals.

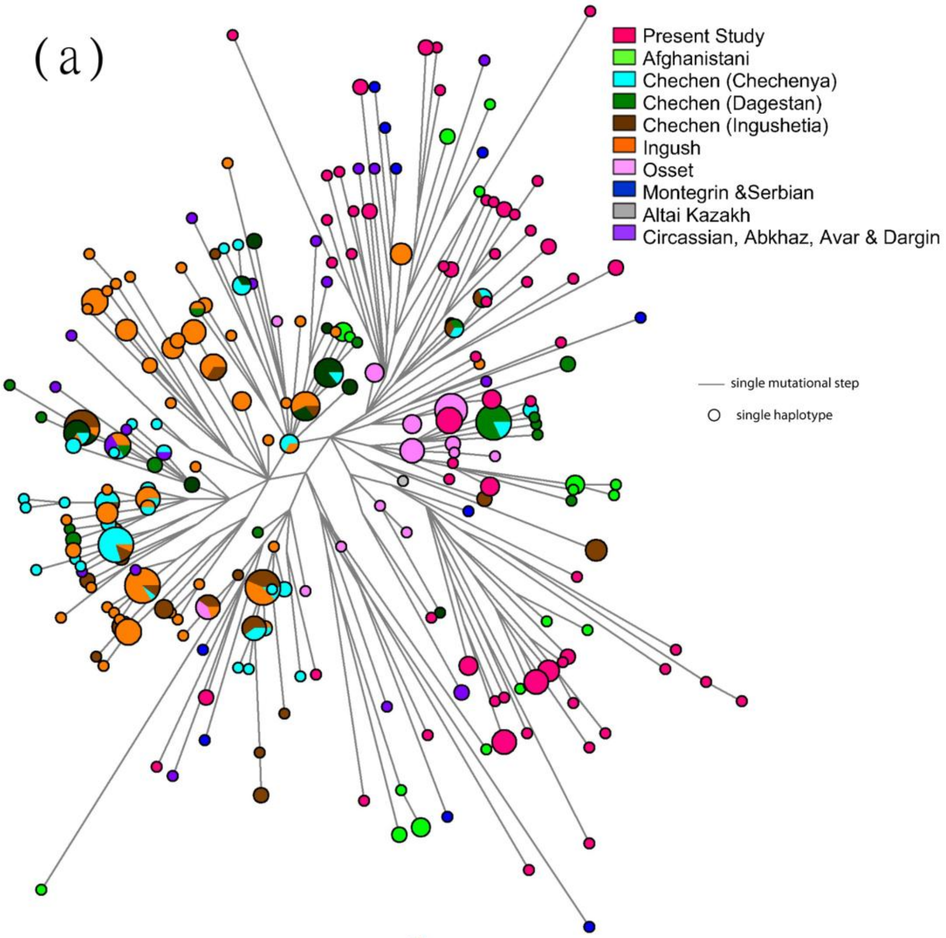

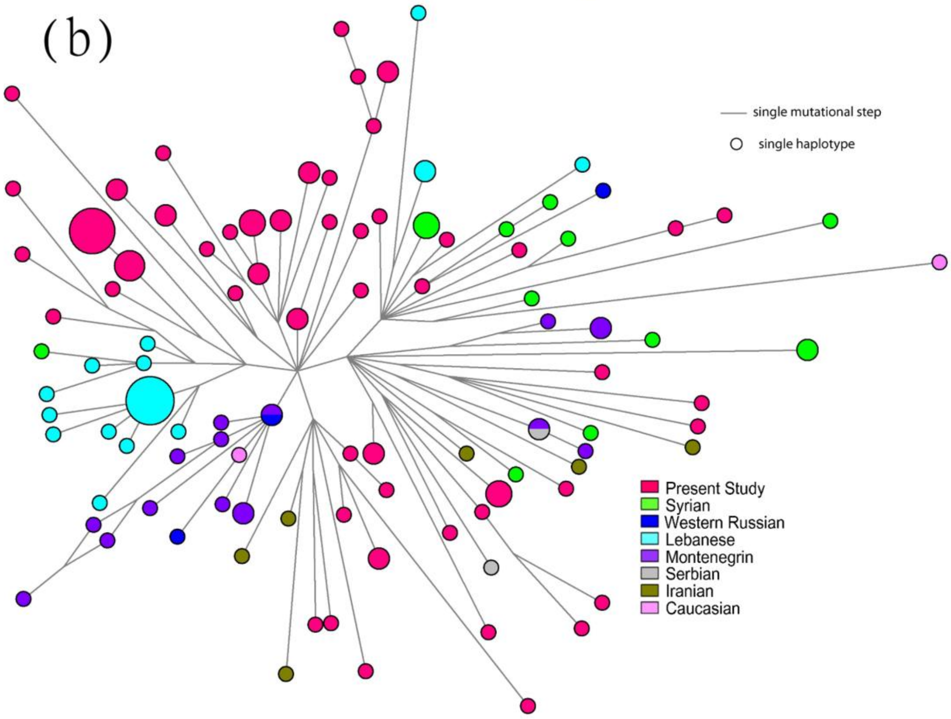

Figure S2. Reduced median phylogenetic network relating Y-STR haplotypes within (a) J2a-M410 & (b) J2b-M102 worldwide. The size of the circle is proportional to the numbers of the samples.

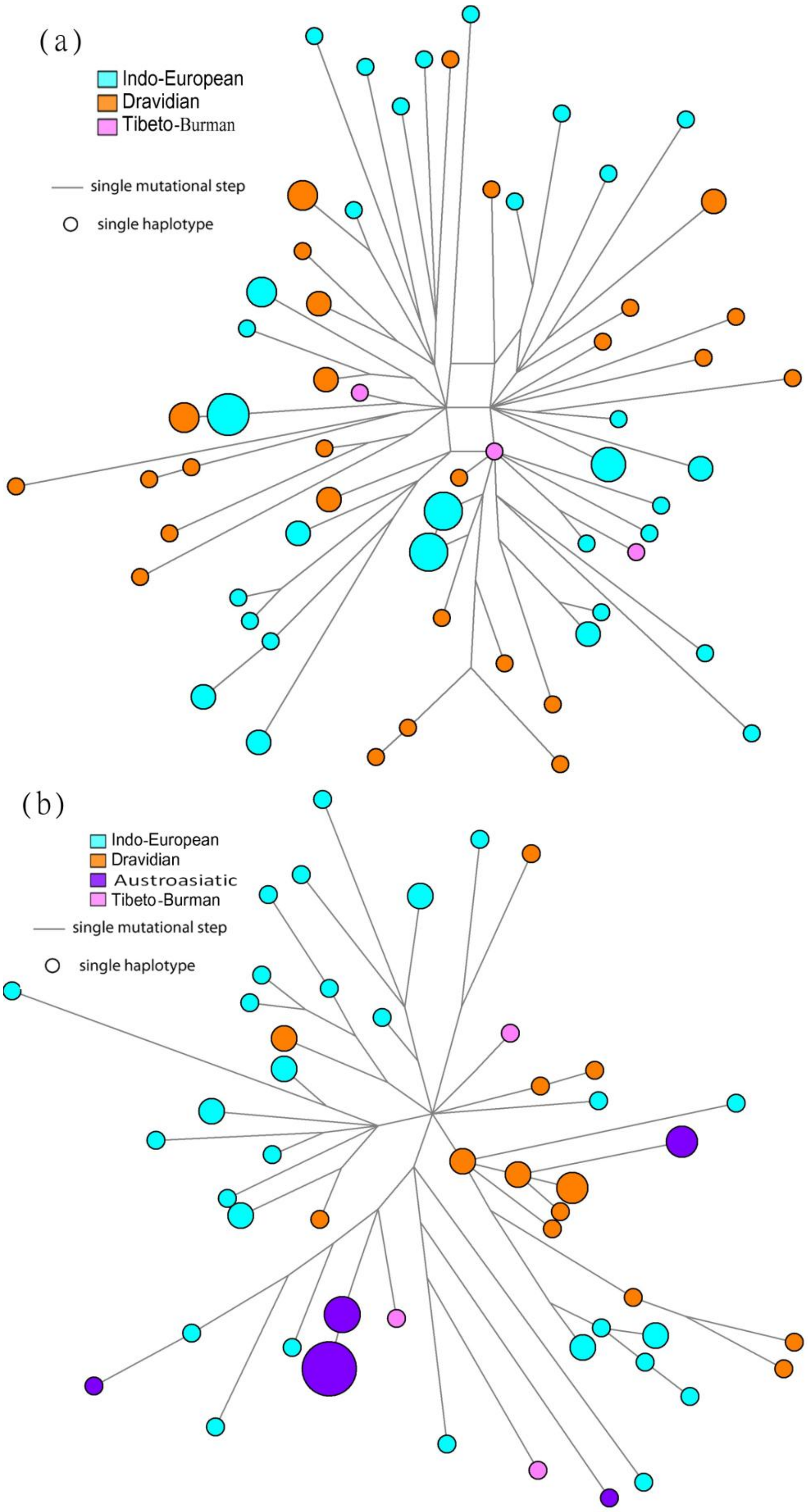

Figure S3. Reduced median phylogenetic network relating Y-STR haplotypes within (a) J2a-M410 & (b) J2b-M102 in India. The size of the circle is proportional to the numbers of the samples.

Supplementary Text S1

Sampling and genotyping:

Blood or buccal swab samples were collected with the informed written consent from 3023 unrelated healthy individuals belonging to 77 populations from all the four linguistic groups of India (Table S2). This project was approved by Institutional Ethical Committee (EIC) of CCMB. The methods were carried out in accordance with approved guidelines. DNA isolation was performed using the standard protocol published elsewhere [1]. Total 355 individuals were found to be J-M304 derived. To amplify the template DNA using 10 pM of each primer, 100 µM dNTPs, 1.5 mM MgCl2 and 1 U of Taq DNA polymerase. Thirty five cycles of reaction were performed with 30 seconds denaturation at 94°C, 30 seconds annealing at 55°C and 2 minutes extension at 72°C. The final extension was done for 7 minute. Sequencing reaction was carried out with BigDye™ Terminator cycle sequencing kit (Applied Biosystems, USA) using ABI 3730XL DNA Analyzer (Applied Biosystems, USA). We compared our results with the published 6966 Y chromosomes belonging from different populations worldwide (Table S1).

Y-STR typing

For Y-STR genotyping one to five J2a-M410 and/or J2b-M102 individuals were randomly selected from each population, based upon the frequencies in the respective population. Total, 158 individuals were typed for loci DYS19, DYS389I and II, DYS385, DYS390, DYS391, DYS392, DYS393, DYS456, DYS458, DYS437, DYS438, DYS439, DYS448, DYS635 and Y GATA H4 by using the AmpFℓSTR® Y-filer™ PCR amplification Kit (Applied Biosystems) following the conditions (1) 95°C for 11 min, (2) 30 cycles: 94°C for 1 min, 61°C for 1 min, 72°C for 1 min, (3) 60°C for 80 min, and (4) 25°C hold. The PCR amplicons along with GS500 LIZ (as size standard) were run in the ABI 3730XL DNA Analyzer (Applied Biosystems, Foster City, USA). Fragment sizes were determined using the GeneMapper® Analysis Software v4.0 and allele designations were based on comparison with allelic ladders included in the Yfiler™ kit. The alleles were noted for all except changes in the two loci where “DYS389I” was used as “DYS389cd” and “DY389ab” = (DYS389II-DYS389I). Out of 17 loci obtained, two DYS385 loci were excluded from the current analyses. Thus, all the analyses linked with Y-STR data were carried out with 15 loci (Table S3 & S4).

Reference

1. Thangaraj, K., *et al*. CAG repeat expansion in the androgen receptor gene is not associated with male infertility in Indian populations. *J Androl* **23**, 815-818 (2002).
